# Limited Duplication-Based List Scheduling Algorithm for Heterogeneous Computing System

**DOI:** 10.3390/mi13071067

**Published:** 2022-07-03

**Authors:** Hong Guo, Jiayin Zhou, Haonan Gu

**Affiliations:** 1 Hubei Province Key Laboratory of Intelligent Information Processing and Real-Time Industrial System, College of Computer Science and Technology, Wuhan University of Science and Technology, Wuhan 430065, China; guohong@wust.edu.cn (H.G.); chris@wust.edu.cn (H.G.); 2 College of Computer Science, Wuhan Qingchuan University, Wuhan 430204, China

**Keywords:** heterogeneous computing systems, list scheduling, task duplication scheduling, limited duplication, random graph generator

## Abstract

Efficient scheduling algorithms have been a leading research topic for heterogeneous computing systems. Although duplication-based scheduling algorithms can significantly reduce the total completion time, they are generally accompanied by an exorbitant time complexity. In this paper, we propose a new task duplication-based heuristic scheduling algorithm, LDLS, that can reduce the total completion time and maintains a low time complexity. The scheduling procedure of LDLS is composed of three main phases: In the beginning phase, the maximum number of duplications per level and per task is calculated to prevent excessive duplications from blocking regular tasks. In the next phase, the optimistic cost table (OCT) and ranking of tasks are calculated with reference to PEFT. In the final phase, scheduling is conducted based on the ranking, and the duplication of each task is dynamically determined, enabling the duplicated tasks to effectively reduce the start execution time of its successor tasks. Experiments of algorithms on randomly generated graphs and real-world applications indicate that both the scheduling length and the number of better case occurrences of LDLS are better than others.

## 1. Introduction

With the development of cutting-edge techniques, such as big data and machine learning as well as the progressive scaling of application size and complexity, the parallel and distributed computing has been increasingly in the focus of research [1].

Task scheduling remains an important part of the operating system, and the quality of the scheduling algorithm can impact how well the computing system utilizes the processor resources. All types of computing systems need a suitable scheduling algorithm to improve the response time and shorten the total computation time. Heterogeneous multi-core systems are preferable because of their better performance in dedicated computing. The heterogeneous platform consists of multiple single-core or multi-core processors of different kinds, for example, the more common heterogeneous multi-core architecture usually consists of a multi-core processor CPU and GPU.

The task scheduling problem of heterogeneous computing systems is more complex than that of homogeneous systems. The computation time of a task on each processor varies from the hardware platform, as well as the communication rate between different kernels. A directed acyclic graph (DAG) is commonly used to represent the tasks pertaining to applications. Studies have shown that DAG task scheduling is an NP-complete problem [2], where an optimal solution for scheduling cannot be found in polynomial time. Therefore, within the realm of research on scheduling, much of the focus has been on studying low time complexity heuristics to obtain a better solution [3]. The total completion time of a task or makespan is often used as an important scheduling result to measure the merit of scheduling algorithms.

Duplicating tasks to the appropriate cores can decrease the data waiting time of their successors and enhance the parallel computation time of heterogeneous systems, thus making fuller use of CPU resources and significantly reducing the total computation time. However, duplication-based scheduling strategies have drawbacks. The complexity of duplication-based algorithms is generally higher, and duplication also causes more computer power consumption. Improper replicate strategies also obstruct the execution of other tasks, causing problems such as makespan expansion.

In this paper, we propose a novel duplication scheduling strategy that limits the maximum number of duplications per level and per task, which makes dynamic decisions on duplication during the scheduling process. Based on these heuristic limits, the duplication of tasks will not be too excessive so as to block the execution of subsequent tasks, and it can also effectively minimize the total completion time of all tasks and improve the utilization of processors.

In this paper, we combined our proposed duplication policy with the list scheduling algorithm predict earliest finish time (PEFT) [3], and used its proposed optimistic cost table (OCT) as well as ranking for scheduling in the scheduling phase of each task; After that, it enters the duplication phase and selects the appropriate number of duplications for each task according to our duplication limitation policy with effective duplication processors. Afterward, our algorithm is experimentally validated to achieve better results compared to both PEFT and other duplication-based scheduling algorithms. It also maintains the same time complexity as PEFT.

The rest of the paper consists of the following sections: In the next section, we present studies related to DAG task scheduling under heterogeneous multi-core computing systems, and focus on the task duplication-based scheduling algorithm. In Section 3, we describe the basic model of scheduling, related parameters, and formulas. Section 4 introduces the details of the implementation of our proposed algorithm. Section 5 illustrates the comparison data of our proposed algorithm with multiple scheduling algorithms on a series of randomly generated task graphs. The summary and outlook of this paper are written and given in the last section.

## 2. Related Works

This section briefly introduces the study of scheduling algorithms in heterogeneous computing systems, focusing on list scheduling algorithms and duplication-based scheduling algorithms.

Task scheduling algorithms can be broadly classified into two categories: static scheduling and dynamic scheduling. Static scheduling means that all required data, such as the heterogeneous execution time of all tasks, communication time of data, and task dependencies (predecessor and successor tasks), are known before scheduling is performed. With static scheduling, a definite scheduling scheme can be derived, and will not actually execute until the exact scheduling of each task is calculated.

As, for dynamic scheduling, such information is unknown or partially available, tasks are dynamically scheduled, and its scheduling decisions are not all made in the beginning [3,4]. While dynamic scheduling requires less information and permits arbitrary tasks to join, static scheduling yields a better scheduling result by evaluating all tasks, and it has no runtime overhead [4,5].

In this paper, we consider static scheduling algorithms because of their efficiency in planning scheduling arrangements, which could reduce the total completion time of tasks. Static scheduling algorithms can be divided into two categories: heuristic-based and guided random search-based algorithms. The former can be classified into three categories: list scheduling, cluster scheduling, and task duplication scheduling [6]. The heuristic methods have been widely studied for their capacity to find suboptimal solutions for scheduling at a lower time complexity [7].

### 2.1. List Scheduling

List scheduling algorithms require maintaining a list containing all the tasks in a sequence that is arranged in fixed priority order. These schedules consist of two main phases: the priority calculation phase and the scheduling phase [3]. The task with the highest-ranking priority will be scheduled first, and the scheduling phase will assign the task to the most appropriate processor to run according to the policy which reduces the overall scheduling time [8]. The priority calculation and scheduling strategies vary from one list scheduling algorithm to another.

The classical algorithm heterogeneous earliest finish time (HEFT) [6] has defined an upward ranking method that recursively calculates the priority of each task based on its average execution time, communication time, and the ranking of the precursor tasks. Then the scheduling tasks are arranged in a non-increasing order of their priorities.

In the HEFT scheduling phase, the processor which brings the task to finish earliest is directly selected for scheduling.The time complexity of the HEFT algorithm is $O(v^{2}\cdot p)$. In the article [9], a variety of heuristic heterogeneous scheduling algorithms were evaluated, and HEFT was concluded to be optimal in terms of reliability and scheduling length at that time. Since HEFT has lower complexity and better scheduling results, it is frequently used as a reference for comparison with other algorithms [7].

The lookahead algorithm [10] makes improvements to the processor selection strategy based on HEFT. It proposes four prospective methods, but all of them are required to calculate the earliest completion time of all subtasks of the current scheduling task in order to decide the optimal processor. Although it can obtain superior scheduling results compared to HEFT, it drastically increases the time complexity of the algorithm to $O(v^{4}\cdot p^{3})$ [3,10].

The more recent algorithm predict earliest finish time (PEFT) [3] defines the optimistic cost table (OCT), which is used to prioritize and schedule tasks. It outperforms such algorithms as HEFT and lookahead [3] in terms of scheduling length ratio (SLR) and completion time while maintaining the same low time complexity $O(v^{2}\cdot p)$ as HEFT.

### 2.2. Duplication-Based Scheduling

The central concept of duplication-based scheduling algorithms is to make redundant copies of tasks to start succeeding tasks earlier and to reduce the influence of inter-processor communication time [6]. The main differences between duplicate scheduling algorithms are as follows: task priority ranking, processor selection policy, and task duplication policy.

Several early duplication-based scheduling algorithms are critical path fast duplication (CPFD) [11], duplication scheduling heuristic (DSH) [12], bottom-up top-down duplication heuristic (BTDH) [13], etc.

The policy of CPFD is to copy $n_{p}$ to processor *J* if task $n_{i}$ has a parent $n_{p}$ on the critical path, and $n_{p}$ is not on the processor *J* to which $n_{i}$ will be scheduled, and if copying it to that core would not delay the execution of $n_{i}$. The DSH algorithm tries to copy the precursor task to be scheduled into the free time slot of the current processor until the time slot is depleted or the task’s EFT ceases to decrease, and repeats the process for other processors. The BTDH algorithm makes an improvement to the DSH algorithm by its continuing to replicate the predecessor task, even when the duplication time slot is filled and the earliest start time of the task is temporarily increased, where the complexity of DSH and BTDH reaches $O(v^{4})$ [11,12,13,14].

After the publication of the HEFT algorithm, some researchers added duplication strategies on top of it, such as heterogeneous earliest finish with duplicator (HEFD) [15], which iterates through all processors when scheduling task $n_{i}$, calculates the data arrival time DAT of $n_{i}$ direct parent task $n_{j}$ in a non-increasing order, and duplicates the start point of $n_{i}$ if it can be earlier. Its complexity is nearly $O(v^{3}\cdot p)$.

The recent duplication algorithms such as the duplication and insertion algorithms based on list scheduling (DILS) [16] have a larger time complexity $O(v^{4}\cdot p^{2})$ due to the high depth of loops and the excessive computation of the strategy. Some algorithms also argue that traditional duplication strategies greatly enlarge the processor loads and increase energy consumption [8]. List scheduling with task duplication (LSTD) [8] replicates only the entry tasks in order not to over-copy the tasks, which can achieve better duplication results without increasing the processor loads.

## 3. Scheduling Problem

In order to tackle the scheduling problem of heterogeneous multi-core systems, a general scheduling model is commonly used to simplify the problem, and simulation scheduling algorithms are built on top of it to study the feasibility of scheduling. The scheduling algorithm in this paper is also based on this model.

In the task scheduling model, an application can be regarded as multiple dependent tasks, and since tasks need to be executed successively according to their dependencies, it can be represented by a directed acyclic graph (DAG), G=(V,E), where *V* denotes the set of *n* tasks, and each $v_{i}\in V$ denotes a task; *E* denotes the set of graph edges, and each of its edges $e_{i,j}\in E$ denotes the dependencies between tasks.

In a heterogeneous computing system, the execution time of a task on different processors varies. The model defines a v×p matrix *W* to store the execution time of a task on each core, *p* represents the number of processors, and $w_{i,j}$ denotes the execution time of task $v_{i}$ on $p_{j}$ processor.

**Definition** **1.** *$pred(n_{i})$: denotes the set of direct predecessors of task $n_{i}$ in the DAG graph. The entry task of the DAG task graph is indicated as $n_{entry}$, which has no predecessors.*

**Definition** **2.** *$succ(n_{i})$: indicates the set of direct successor tasks of task $n_{i}$ in the DAG graph. The exiting task is represented as $n_{exit}$.*

When multiple tasks represented as DAG graphs appears, or the exit task is not unique, a pseudo-task node with zero computation time and transmission time will be added as an entry or exit node, thus improving the universality of the algorithm.

**Definition** **3.** *$\bar{w_{i}}$: in Equation (1), denotes the average computation time of the ith task on all cores, which is used by many algorithms to compute the priority, for example, HEFT uses this property to evaluate the ranking. The $w_{i,j}$ denotes the execution time of task i on the jth core.*

(1) $$\bar{w_{i}}=\sum_{j=1}^{p}w_{i,j}/p$$

For each edge $e_{i,j}\in E$, it corresponds to a non-negative communication time $c_{i,j}$, indicating the time consumed when the task $v_{i}$ needs to transfer data to another core to execute its direct successor task $v_{j}$.

**Definition** **4.** *$\bar{c_{i,j}}$: in Equation (2), denotes the average communication time between task $v_{i}$ and task $v_{j}$. The formula $\bar{L}$ denotes the average latency of all processors and $\bar{B}$ denotes the data transfer bandwidth of P processors linked to each other. $Data_{i,j}$ denotes the amount of data to be transferred.*

(2) $$\bar{c_{i,j}}=\bar{L}+\frac{Data_{i,j}}{\bar{B}}$$

It is noted that there is no communication consumption if the successor task $v_{j}$ is executed on the same core. To simplify the problem, in this paper, we take the latency to be zero and the data link bandwidth between each processor to be equivalent.

**Definition** **5.** *$level(n_{i})$: in Equation (3), indicates the level at which the task $n_{i}$ is positioned, being the maximum value of the number of path edges from the entry node to itself. It can be expressed by the following formula, and the duplication restriction policy on which this paper is based will consider the number of tasks per level. Provided that $level(n_{entry})=0$.*

(3) $$level\left(n_{i}\right)=\max_{q\in pred(n_{i})}\left\{level\left(q\right)\right\}+1$$

**Definition** **6.** *$EST(n_{i},p_{j})$: in Equation (4), denotes the earliest start time of task $n_{i}$ on processor $p_{j}$, where $T_{Available}\left(p_{j}\right)$ is the processor’s ready time and $AFT(n_{m})$ is the actual finish time of the task. The communication time $c_{m,i}$ is zero if the precursor task $n_{m}$ is scheduled exactly on processor $p_{j}$.*

(4) $$EST\left(n_{i},p_{j}\right)=\max\left\{T_{Available}\left(p_{j}\right),\max_{n_{m}\in pred(n_{i})}\left\{AFT\left(n_{m}\right)+c_{m,i}\right\}\right\}$$

**Definition** **7.** *$EFT(n_{i},p_{j})$: in Equation (5), denotes the earliest finish time of task $n_{i}$ on processor $p_{j}$, which is the earliest start time of that task $EST(n_{i},p_{j})$ plus its computation time $w_{i,j}$ on that processor.*

(5) $$EFT\left(n_{i},p_{j}\right)=EST\left(n_{i},p_{j}\right)+w_{i,j}$$

**Definition** **8.** *$DAT(n_{i},n_{j})$: in Equation (6), the data arrival time is the earliest point in time at which a particular successor task could have started. As shown in the formula, the earliest start time of the successor task $n_{j}$ to task $n_{i}$ is its end time plus the communication time. $AFT(n_{i})$ is the actual finish time of the scheduled task $n_{i}$. The case used for evaluating duplication does not consider that $n_{i},n_{j}$ are executed on the same core and that the task $n_{i}$ is scheduled before evaluating duplication, and its real completion time $AFT(n_{i})$ is known.*

(6) $$DAT\left(n_{i},n_{j}\right)=AFT\left(n_{i}\right)+c_{i,j}$$

Since DAT is used to measure the direct successor of task $n_{i}$, the other parameter needs to be satisfied with $n_{j}\in succ(n_{i})$.

## 4. Proposed Algorithm

In this section, we propose several universal limited duplication strategies and use them for list scheduling, which we call the limited duplication-based list scheduling algorithm (LDLS).The LDLS algorithm has three steps: the computation of the copying limitation table, the calculation of the task priority, and the task scheduling phase.

In the task scheduling phase, our policy not only restricts duplication based on the previously computed table, but also relies on the dynamic minimum data transfer time for duplication judgments. Compared with other duplication algorithms that consider critical paths [11], most important precursor tasks [12,13,17], and idle gaps [12,13], our proposed duplication strategy is heuristic and also maintains a low time complexity consequently.

### 4.1. Duplication Strategies

Excessive duplication can cause blocking the normal execution of tasks at this level, e.g., a task is copied to many cores so that the execution of tasks at the same level of the DAG graph that could run in parallel on multiple cores is delayed. Useless tasks can also be generated in terms of the number of tasks whose replication is finished, exceeding the number of their direct successors. All these problems lead to resource wastage and an increase in finish time. In this case, we propose multiple schemes to limit duplication without increasing the time complexity of the algorithm.

Tasks at the same level in the DAG graph often have time intersections running in parallel, and too many copies of a task may block the timely execution of other tasks at the same level. Therefore, we propose a limitation policy to calculate the maximum number of duplication DP(level) for a task at a particular level based on the number of processors and the number of tasks, as shown in the following equation.

(7) $$DP\left(level\right)=num\left(P\right)-\sum_{n_{i}\in V_{level}}n_{i}$$

Another limitation policy applies to each task. The number of a task in the processor cannot exceed the number of tasks that follow the task $n_{i}$; otherwise, the copied tasks $n_{i}$ would be pointless and may jam other tasks. Therefore, we define $TDP(n_{i})$ to store the maximum number of duplicates of $n_{i}$, given by the following formula. We subtract one because the task itself already occupies a processor.

(8) $$TDP\left(n_{i}\right)=\sum_{j=1}^{x}n_{j}-1,n_{j}\in succ(n_{i})$$

These two limitations could ensure the normal execution of irreplicated tasks to a greater extent, and a dynamic duplication limitation policy is proposed in the subsequent sections. The DP,TDP values are also dynamically changed during the subsequent duplication and scheduling phases. The computation of duplication limitations requires to traverse the edge table once, so the time complexity is O(v+e) when the DAG graph is stored in the adjacency table and $O(v^{2})$ when it is stored in the adjacency matrix.

### 4.2. Task Priority

The algorithm proposed in this paper borrows the optimistic cost table OCT introduced by PEFT [3] for the priority calculation. The size of OCT is a matrix of size v×p in the same way as the task time consumption matrix *W*. $OCT(t_{i},p_{k})$ denotes the maximum value of the shortest path length of the successor tasks of $t_{i}$ when the task $t_{i}$ is assigned to processor $p_{k}$. It is recursively calculated by Equation (9).

(9) $$OCT\left(t_{i},p_{k}\right)=\max_{t_{j}\in succ(t_{i})}\left[\min_{p_{w}\in P}\left\{OCT\left(t_{j},p_{w}\right)+w\left(t_{j},p_{w}\right)+\bar{c_{i,j}}\right\}\right]$$

The formula calculates the minimal finishing time of all succeeding tasks for $t_{i}$ recursively, the maximum of which can be a useful evaluation of the computation time of task $t_{i}$. The $\bar{c_{i,j}}$ refers to Equation (2). For the export task, $OCT(n_{exit},p_{*})=0$. The task ranking is calculated from the OCT table by taking the average OCT of the task over all cores, as shown in Equation (10).

(10) $$rank_{oct}\left(t_{i}\right)=\frac{\sum_{k=1}^{P}OCT\left(t_{j},p_{k}\right)}{P}$$

### 4.3. Dynamic Duplication Scheduling

In the scheduling phase, instead of scheduling based on the earliest finish time EFT in Equation (5), regarding the PEFT implementation, the task is assigned to the core that makes it the smallest by calculating the $O_{EFT}$ of the task on each core with reference to the OCT table. The formula is as follows (Equation (11)).

(11) $$O_{EFT}\left(t_{i},p_{j}\right)=EFT\left(t_{i},p_{j}\right)+OCT\left(t_{i},p_{j}\right)$$

In many duplication-based scheduling algorithms, duplication is performed only when all succeeding data arrival time (DAT) is greater than the finish time of the duplication on the core $p_{i}$. Any task that makes the DAT smaller causes copying to be skipped.

The actual DAT of the task that can start earliest among all succeeding tasks is taken to be a fixed value when traversing whether duplication is performed on each core for task $n_{i}$, and there may be cases where the same task causes multiple cores to be judged as non-replicating tasks when that one task only needs to be scheduled once, which can result in insufficient duplication.

To solve this problem, we propose a dynamic minDAT policy by adding an auxiliary array to store tasks that have an earlier data arrival time than the finish time of the duplicated task, as shown in Equation (12). The calculation of $DAT(n_{i},n_{j})$ refers to Equation (6).

In this way, if task $n_{j}$ starts executing faster since it can transfer data across cores, it is assumed that it will be scheduled to this core (not always in practice), the duplication of task $n_{i}$ on this core is skipped, and $n_{j}$ is added to skipped; this task is not considered in later $minDAT(n_{i})$ calculations.

(12) $$minDAT\left(n_{i}\right)=\min\left\{DAT(n_{i},n_{j})\right\},n_{j}\in succ(n_{i})\&n_{j}\notin skipped$$

The complete scheduling algorithm LDLS pseudocode is given in Algorithm 1:
**Algorithm 1** The LDLS algorithm.1:Compute DP,TDP using Equations (7) and (8).2:Calculate OCT table and $rank_{oct}$ for all tasks using PEFT’s Equations (9) and (10).3:Create an empty list ready-list and put $n_{entry}$ as initial task.4:**while**ready-list is not empty **do**5:    $n_{i}\leftarrow$ Task popped from ready-list which has the highest $rank_{oct}$.6:    **for** processor $p_{i}$ in processor-set *P* **do**7:         Compute $EFT(n_{i},p_{i})$ with Equation (5) and PEFT’s $O_{EFT}\left(n_{i},p_{i}\right)$ with Equation (11).8:    **end for**9:    Assign task $n_{i}$ to the processor $p_{best}$ which minimize $O_{EFT}$ value.10:  **if** $DP\left[level(n_{i})\right]>0$ and $TDP(n_{i})>0$, $level(n_{i})$ in Equation (3) **then**11:       Create empty list skipped to record task that has less DAT, Equation (6).12:       **for** processor $p_{d}$ in processor-set $P-p_{best}$ **do**13:          Calculate $EFT(n_{i},p_{d})$, and $minDAT(n_{i})$ using Equation (12).14:           **if** $EFT\left(n_{i},p_{d}\right)\geq minDAT(n_{i})$ **then**15:               Add $n_{min}$ that minimize $minDAT(n_{i})$ to skipped.16:               Reduce $TDP(n_{i})$ by 1, then continue loop.17:           **else**18:               Duplicate task $n_{i}$ on processor $p_{d}$.19:               Reduce $DP\left[level(n_{i})\right]$ and $TDP(n_{i})$ by 1.20:           **end if**21:           Once $TDP(n_{i})<1$, break task duplicate loop.22:       **end for**23:   **end if**24:   Update ready-list with $n_{j}\in succ(n_{i})$ if $n_{j}$ not in ready-list.25:**end while**

The algorithm first calculates the required DP,TDP,OCT table, and $rank_{oct}$, putting the entry tasks into the ready-list. The task with the highest priority in the ready-list is scheduled in the while loop.

The scheduling phase starts in the sixth line, which calculates the EFT and $O_{EFT}$ of the task on each core, schedules the task $n_{i}$ on processor $p_{best}$, which minimizes $O_{EFT}$. Starting from the tenth line, the duplication phase is entered only if the number of $DP[level\left(n_{i}\right)]$ and $TDP(n_{i})$ are both bigger than zero.

First, an empty array skipped is created to hold the succeeding tasks $n_{j}$ that have skipped duplication.

Then it iterates through each processor (except the previously scheduled core $p_{best}$) in line 12 and computes the earliest finish time of the task on the core to be copied and the minimum data arrival time in the current skipped state. Note that the order of iterations in line 12 is in increasing order of the time consumption of tasks on different processors (e.g., the first processor selected is the one with the shortest time to complete the task).

If there is a situation where the data arrive earlier than the duplicated task finishes, as mentioned before, this task is put into the skipped array and reduce the number of task duplications by one; otherwise, the task is duplicated on that processor and the number of replicable times is reduced for both that layer and that task by one.

Once the number of available copies of a task goes to zero, the algorithm jumps out of the duplication phase. After ending the duplication phase, the succeeding tasks $succ(n_{i})$ are added to the ready-list.

In terms of time complexity, computing the DP,TDP table only requires traversing the edge table once, and the time complexity of DAG graphs is O(v+e) in the case of the adjacency table represented task; it is $O(v^{2})$ in the case of adjacency matrix representation. The OCT is computed as O(p(e+v)), which can be considered $O(v^{2}\cdot p)$ when the DAG graph is represented by the adjacency matrix. The task duplication phase of the scheduling process is at the same level as the loop unit of the scheduling phase, and the time complexity is both $O(v^{2}\cdot p)$. Therefore, the total time complexity of LDLS is $O(v^{2}\cdot p)$, which is consistent with the algorithm PEFT and HEFT.

## 5. Experimental Results

This section evaluates our proposed LDLS algorithm against PEFT [3], LSTD [8], and HEFT [6], with respect to each comparison metric. Firstly, we introduce the comparative metrics to measure the performance of the algorithm. In order to make a more comprehensive and detailed evaluation of the algorithm, we also use a variety of parameters for the random generation of the task graph. Likewise, we evaluate real-world tasks too. Finally we present the comparison results for each parameter.

### 5.1. Comparison Metrics

When evaluating the performance of an algorithm, the scheduling length of the graph (or makespan) is the fundamental criterion for evaluation, on top of which there is a pairwise comparison of the number of occurrences of better solutions among algorithms and the scheduling length ratio. Due to the long scheduling time of large task graphs, ref. [6] proposed a method to calculate the scheduling length normalized to a smaller range. This method is called the scheduling length ratio (SLR), and it is defined by the following Equation (13).

(13) $$SLR=\frac{makespan}{\sum_{n_{i}\in CP_{MIN}}\min_{p_{j}\in P}\left\{w_{i,j}\right\}}$$

The denominator of the formula is the sum of the minimum execution time of the tasks on the path $CP_{MIN}$ ($CP_{MIN}$ refers to the critical path of the graph considering all tasks execution time as the minimal value of the task). As this critical path may not all reach the minimum computation time on a single core, its value is only used as the theoretical minimum computation time, which is practically impossible to reach; therefore, SLR is greater than 1, and the smaller the number, the better the scheduling result.

The rate of occurrences of better solutions metric is compared in pairs of algorithms based on scheduling makespan, and the percentage of better, equal, and worse solutions for a given algorithm are presented in the pairwise comparison table.

### 5.2. Random Generated Task Graphs

In this section, we introduce the random graph generator method and its parameters, and make an evaluation analysis of the algorithm.

#### 5.2.1. Random Graph Parameters

We conducted scheduling experiments under nine different parameters in order to gain a comprehensive view of our algorithm for various task graphs. In our experiments, we used the DAG generation procedure provided in [18] to implement five of these parameters to generate DAG graphs of different shapes.

- *n*: the number of nodes in the DAG, which is the number of tasks in the application graph.
- fat: affects the shape of the DAG graph. For a given *n*, the larger fat the more tasks in a single level, leading to a smaller height of the graph, increasing the parallelism of tasks, while smaller values lead to narrow and long DAG graphs (e.g, chain), which will have low parallelism.
- density: determines the number of edges between two levels in the DAG graph. The smaller the value, the smaller the number of edges, and vice versa.
- regularity: the regularity of the node numbers between different levels. A higher value makes the node numbers between the DAG levels more similar, a lower value makes the node numbers between levels more varied.
- jump: determines the maximum number of levels that an edge can jump in the DAG graph. That is, any tasks satisfies $jump>\left[level\left(n_{j}\right)-level\left(n_{i}\right)\right],n_{j}\in succ(n_{i})$.

After generating the different DAG graphs for the above parameters, it is necessary to generate the specified computation and communication consumption. It is also necessary to define the number of processors in the scheduling, etc.

- balance (β): parameter used to assign random task computation time balance, also interpreted as a processor heterogeneity factor, specifying a range of values for $w_{i,j}$ refers to Equation (14). A high β value makes the computation consumption of the same task vary widely across different cores, a lower value means that the task’s computation time is essentially the same across cores. The average computation time $\bar{w_{i}}$ for each task is chosen randomly from a uniform distribution $\left[0,2\times \bar{w_{DAG}}\right]$ (where $\bar{w_{DAG}}$ denotes the average computation time for a given DAG graph), and the β value is then used to determine the computation time for each processor $p_{j}$ on the task computation consumption [6].
  (14) $$\bar{w_{i}}\times \left(1-\frac{\beta }{2}\right)\leq w_{i,j}\leq \bar{w_{i}}\times \left(1+\frac{\beta }{2}\right)$$
- CCR (Communication to computation ratio): ratio between the sum of the weights of edges and nodes in task graph.
- processor: number of processors, the more processors the stronger the parallel computing capability.
- round: the number of scheduling for the same parameter. Since the specific values such as $w_{i,j}$ and $c_{i,j}$ are randomly chosen for each scheduling of the algorithm set, different values will lead to different scheduling results. More rounds are performed for the same parameter scheduling; better results will measure the average performance and reduce the influence of exceptions.

#### 5.2.2. Performance of Random Graphs

The parameters in Listing 1 were selected for our experiments to generate random task graphs for evaluation.

**Listing**  **1.** The parameters for random graph generator.n = [10, 20, 30, 40, 50, 60]fat = [0.1, 0.4, 0.7]density = [0.2, 0.5, 0.8]regularity = [0.2, 0.5, 0.8]jump = [2, 4, 7]CCR = [0.5, 1, 4, 8, 10, 20]β = [0.5, 1, 1.5]prosessor = [8, 16, 32]round = 2

The combination of these parameters produces a total of 52,488 different task graphs, and the experiments perform scheduling experiments on LDLS, PEFT, LSTD, and HEFT for each graph separately, and compute SLR to generate comparison graphs according to Equation (13).

**Figure 1:**
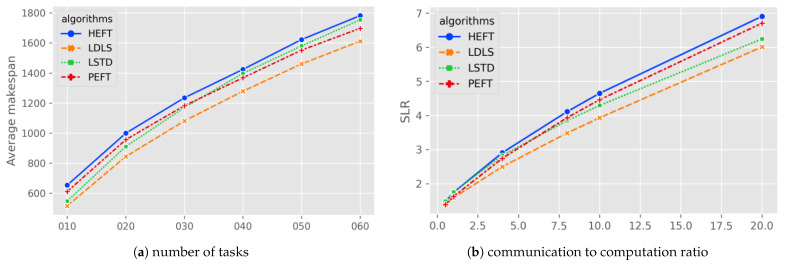
Average makespan/SLR for random graphs as a function of task numbers and CCR.

Figure 1a shows the average makespan comparison of scheduling for different number of tasks. The LDLS algorithm reduces the scheduling length over LSTD (7.59%,8.63%,7.58%,8.10%), over PEFT (8.54%,6.80%,6.43%,5.54%), and over HEFT (12.43%,10.18%,10.00%,9.53%) for the number of tasks 30, 40, 50, 60. In the case of a low number of tasks (n = 10), the LDLS algorithm does not improve much on the LSTD algorithm.

Figure 1b shows the comparison between algorithms at different CCRs. LDLS and PEFT have similar scheduling results at lower CCRs; as CCR increases, LDLS performs better than PEFT and HEFT, and LSTD is better than PEFT and close to LDLS at high CCRs. The LDLS algorithm outperforms the HEFT, PEFT, and LSTD algorithms in the overall cases.

**Figure 2:**
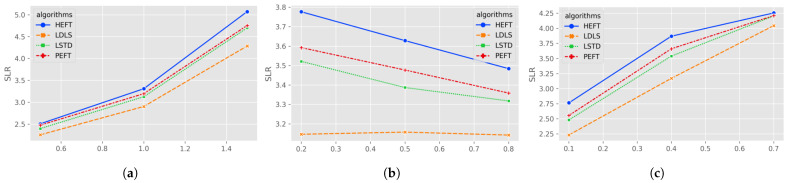
Average SLR for random graphs of (**a**) heterogeneity of processors; (**b**) density; (**c**) fat.

Figure 2 shows the result statistics of the algorithm under different heterogeneity, density and fat. In these cases, LSTD is slightly better than the PEFT algorithm, but both are worse than the LDLS algorithm. In the case of heterogeneity (0.5, 1, 1.5), LDLS improves over LSTD by (5.79%, 7.68%, 9.91%); in the case of density (0.2, 0.5, 0.8), LDLS improves over LSTD by (10.61%, 7.32%, 5.96%); in the case of different fat (0.1, 0.4, 0.7), LDLS improves over LSTD by (10.08%, 10.44%, 4.11%).

**Figure 3:**
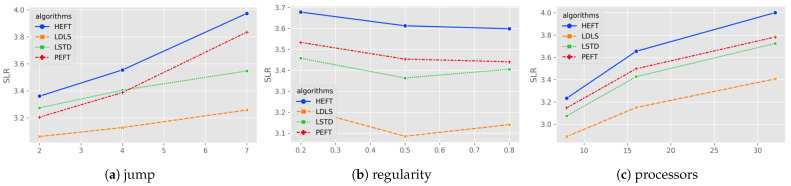
Average SLR for random graphs of (**a**) maximum jump between level; (**b**) regularity of DAG; (**c**) number of processors.

Figure 3 shows the comparison of algorithms on different maximum jumps, regularity and number of processors. The improvement of LDLS on LSTD for maximum jumps of (2, 4, 7) is (6.94%, 8.14%, 8.17%), and the average result of LSTD is worse than PEFT for low jumps, but better than PEFT for high jumps, yet still worse than LDLS. LDLS is better than LSTD (10.02%, 7.32%, 5.96%) at different densities (0.2, 0.5, 0.8). LDLS is better than LSTD for different number of processors (8, 16, 32) with (6.21%, 8.07%, 8.92%).

It is obvious that the LDLS algorithm has a better performance than the other algorithms in the case of high anisotropy, low density, low-fat values, and so on. Averagely, the LDLS has a better performance than the rest of the algorithms.

Table 1 presents the percentage of makespan better, equal, and worse results for the comparison of algorithms. LDLS has 54.17% cases where the scheduling length of the task is shorter compared to PEFT. Since our algorithm is limited duplication, it falls back to PEFT when the duplication condition is not satisfied, so the scheduling results are consistent with it in 35.33% of cases. For LSTD and HEFT, the LDLS algorithm has a better solution in (75%, 76%) cases, respectively.

### 5.3. Real-World Application Graphs

In addition to randomly generated task graphs, we considered the performance of the algorithm in real-world applications. We use Gaussian elimination [3,6,19,20], fast Fourier transform [3,6] and montage [3,8,21] as the basic graphical framework, and generate task graphs with different weights based on the parameters CCR, β and the processor numbers, again repeated six times in each pair. We choose CCRs ranging from [0.1,0.5,0.8,1,2,5] for high-speed networks (CCR = 0.1) to slower networks (CCR = 5); processor heterogeneity β included in [0.2,0.5,1,1.5,1.8] from low processor heterogeneity (β = 0.2) to high heterogeneity; processor number [8,16,32].

#### 5.3.1. Gaussian Elimination

**Figure 4:**
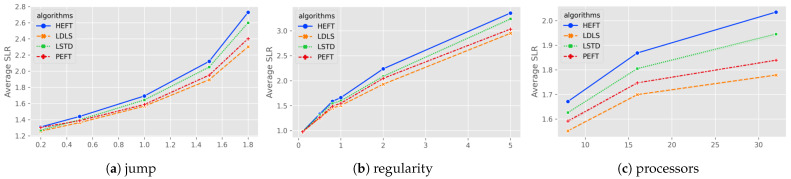
Average SLR for Gauss elimination as a function of (**a**) heterogeneity of processors; (**b**) communication to computation ratio; (**c**) number of processors.

Gaussian elimination corresponds to a realistic task with fine-grained partitioning, whose figure size is defined by a new parameter matrix size *m*, and the number of tasks is equivalent to $\frac{m^{2}+m-2}{2}$. The evaluation results of LDLS, PEFT, LSTD and HEFT for Gaussian elimination maps with different parameters are shown in Figure 4.

For the task graphs with higher heterogeneity (higher β values) (see Figure 4a), the LDLS algorithm improves the LSTD for heterogeneity of (1, 1.5, 1.8) by (4.69%, 7.62%, 11.46%), respectively. Overall, LDLS outperforms the rest of the algorithms for all heterogeneity values.

From Figure 4b, for low communication rate (CCR = 0.1), the scheduling results of the algorithms are generally consistent, and LDLS has an average (5.79%, 7.65%, 8.94%) speedup for LSTD at higher CCR cases (1, 2, 5). For different numbers of processors (8, 16, 32), LDLS has (4.57%, 5.85%, 8.57%) speedup on LSTD. In general, LDLS outperforms LSTD, PEFT and HEFT algorithms (6.45%, 2.86%, 9.77%).

#### 5.3.2. Fast Fourier Transform

**Figure 5:**
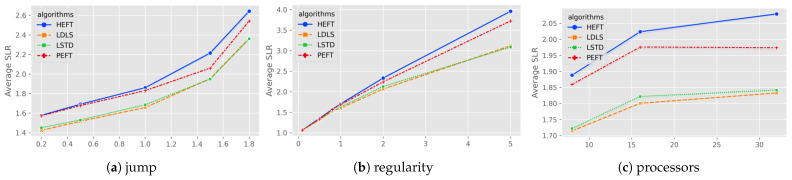
Average SLR for fast Fourier transform as a function of (**a**) heterogeneity of processors; (**b**) communication to computation ratio; (**c**) number of processors.

The scheduling results of LDLS in the fast Fourier transform graph appear to be similar to LSTD. As shown in Figure 5b, the scheduling result of LSTD is a bit better than LDLS at CCR = 5, but the total performance remains that LDLS is better, and the total average speedup obtained by LDLS corresponds to (0.71%, 7.95%, 10.74%) for LSTD, PEFT, and HEFT, respectively. The performance improvement of LDLS in this highly interconnected graph as seen is not significant compared to LSTD, but there is still a sizable improvement for PEFT and HEFT.

#### 5.3.3. Montage Workflow

**Figure 6:**
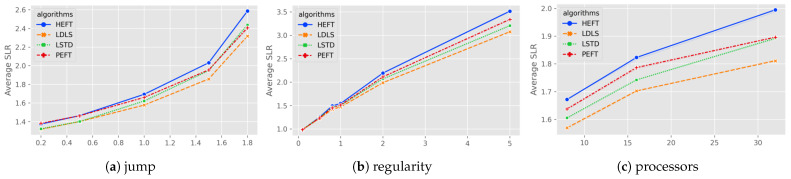
Average SLR for montage as a function of (**a**) heterogeneity of processors; (**b**) communication to computation ratio; (**c**) number of processors.

For the montage graph (see Figure 6), the LDLS algorithm is similar to LSTD in the low heterogeneity case, but as the heterogeneity increases, the LSTD scheduling length discrepancy increases with LDLS ratio gradually. As in Figure 6a, in the high heterogeneity case (1, 1.5, 1.8), LDLS has an average speedup of (2.76%, 4.61%, 4.87%) for LSTD; as in Figure 6b, in the high CCR case (1, 2, 5), LDLS has an average speedup of (2.19%, 3.92%, 4.07%) for LSTD. LDLS has an average speedup of (2.19%, 3.92%, 4.07%). For the rest of the algorithms, total speedups are (2.99%, 4.46%, 7.40%), corresponding to LSTD, PEFT, and HEFT.

## 6. Conclusions

In this paper, we proposed a new heuristic task duplication strategy and used it for the list scheduling algorithm, called limited duplication-based list scheduling (LDLS). This algorithm effectively reduces the total task finish time and improves the processor performance while maintaining the same time complexity $O(v^{2}\cdot p)$ as the classical algorithm PEFT and HEFT.

The LDLS algorithm first generates duplicate limitation tables DP and TDP based on the task and the computing environment, indicating the maximum number of duplicates per level and per task, respectively. Then the priority of the tasks is calculated. Task duplication is heuristically conducted during the scheduling phase dynamically based on the scheduling situation.

Simulated experiments show that the LDLS algorithm outperforms other quadratic time complexity algorithms, such as the classical HEFT and PEFT list scheduling algorithms, as well as the duplication-based LSTD algorithm, in terms of both the scheduling length ratio (SLR) and the frequency of occurrence of more optimal solutions on random graphs.

For real-world applications, our LDLS algorithm has better scheduling results than the duplication algorithm LSTD on Gaussian elimination and montage graphs, while the results are very similar in fast Fourier transform graphs. However, the LDLS algorithm performs better than PEFT and HEFT in all three real-world tasks.

## Figures and Tables

**Table 1:** Pairwise overall makespan comparison of the scheduling algorithms.

		LDLS	PEFT	LSTD	HEFT
LDLS	better		54.17%	75.01%	76.89%
	equal	*	35.33%	5.17%	2.86%
	worse		10.50%	19.82%	20.25%
PEFT	better	10.50%		57.54%	62.23%
	equal	35.33%	*	3.12%	6.26%
	worse	54.17%		39.34%	31.51%
LSTD	better	19.82%	39.34%		49.02%
	equal	5.17%	3.12%	*	8.35%
	worse	75.01%	57.54%		42.63%
HEFT	better	20.25%	31.51%	42.63%	
	equal	2.86%	6.26%	8.35%	*
	worse	76.89%	62.23%	49.02%	

## Data Availability

Data available on request due to restrictions eg privacy or ethical. The data presented in this study are available on request from the corresponding author. The data are not publicly available for privacy reasons.

## Abbreviations

The following abbreviations are used in this manuscript:

LDLS	Limited Duplication-Based List Scheduling
PEFT	Predict Earliest Finish Time
HEFT	Heterogeneous Earliest-Finish-Time
LSTD	List Scheduling with Task Duplication
